# Supplementary material for: Differential Gene Expression Profiles between N-Terminal Domain and Ligand-Binding Domain Inhibitors of Androgen Receptor Reveal Ralaniten Induction of Metallothionein by a Mechanism Dependent on MTF1
Source: Cancers (Basel). 2022 Jan 13;14(2):386. doi: 10.3390/cancers14020386 (PMC8773799; doi:10.3390/cancers14020386)
Supplement: Supplementary file 1 [file cancers-14-00386-s001.zip › cancers-1534137-supplementary.pdf]

Supplementary materials: Differential Gene Expression Profiles between N-Terminal Domain and Ligand-Binding Domain Inhibitors of Androgen Receptor Reveal Ralaniten Induction of Metallothionein by a Mechanism Dependent on MTF1

Jon K. Obst, Nasrin R. Mawji, Simon J.L. Teskey, Jun Wang and Marianne D. Sadar

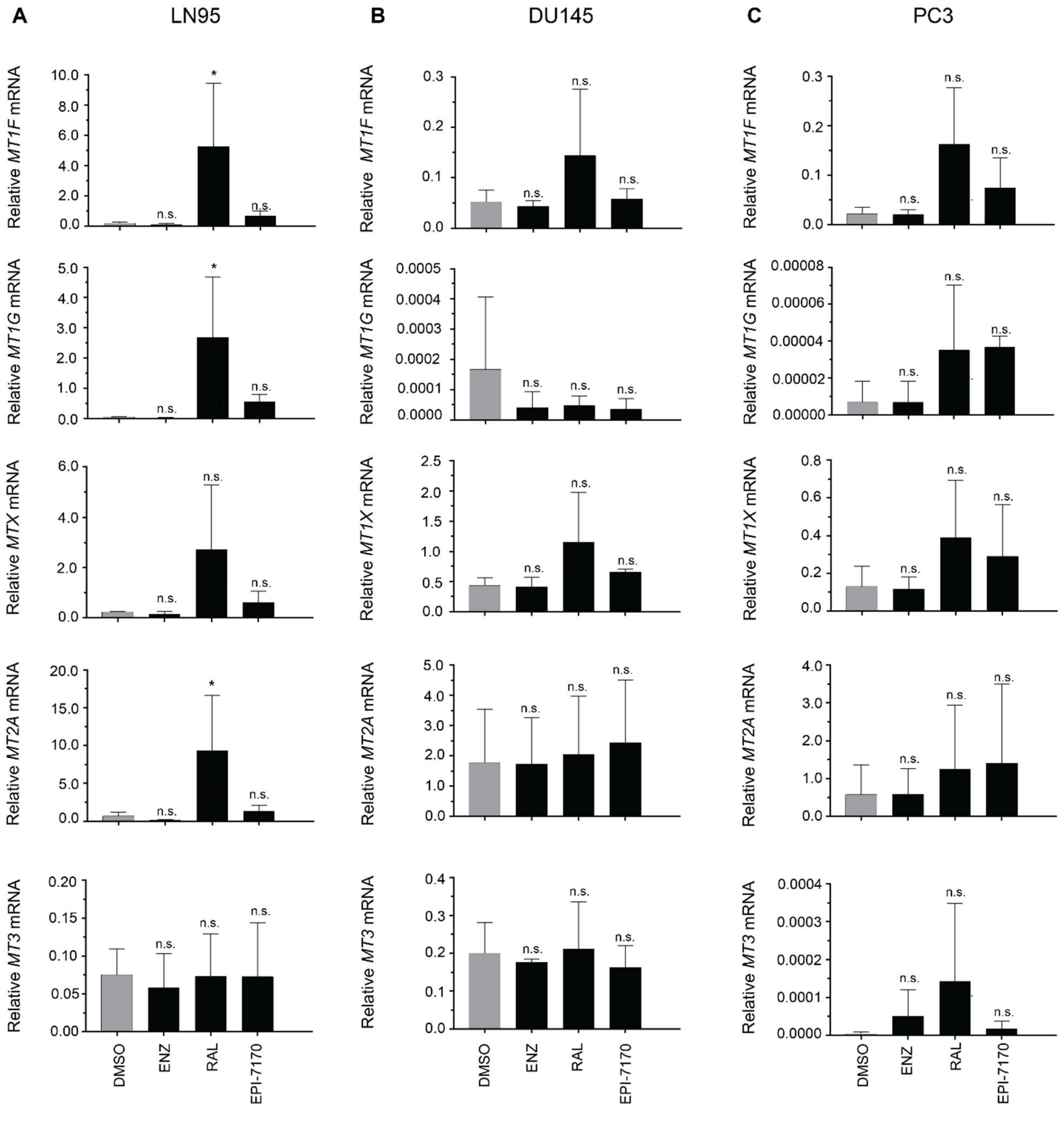

Figure S1. Ralaniten induced metallothionein in prostate cancer cell lines with functional AR. (**A**) Transcript levels of MT1F, MT1G, MT1X, MT2A and MT3 were normalized to levels of SDHA transcript from LN95, (**B**) DU145, (**C**), and PC3 cells treated with enzalutamide (5 μM), ralaniten (35 μM), EPI-7170 (5 μM) or v/v DMSO. Data presented as mean ± SD and analyzed by two-way

ANOVA with Sidak's test applied *post hoc*, $n = 3$ independent experiments. $^{*}p < 0.05$; $^{**}p < 0.01$; $^{***}p < 0.001$; $^{\#}p < 0.0001$; n.s., not significant. ENZ, enzalutamide; RAL, ralaniten.

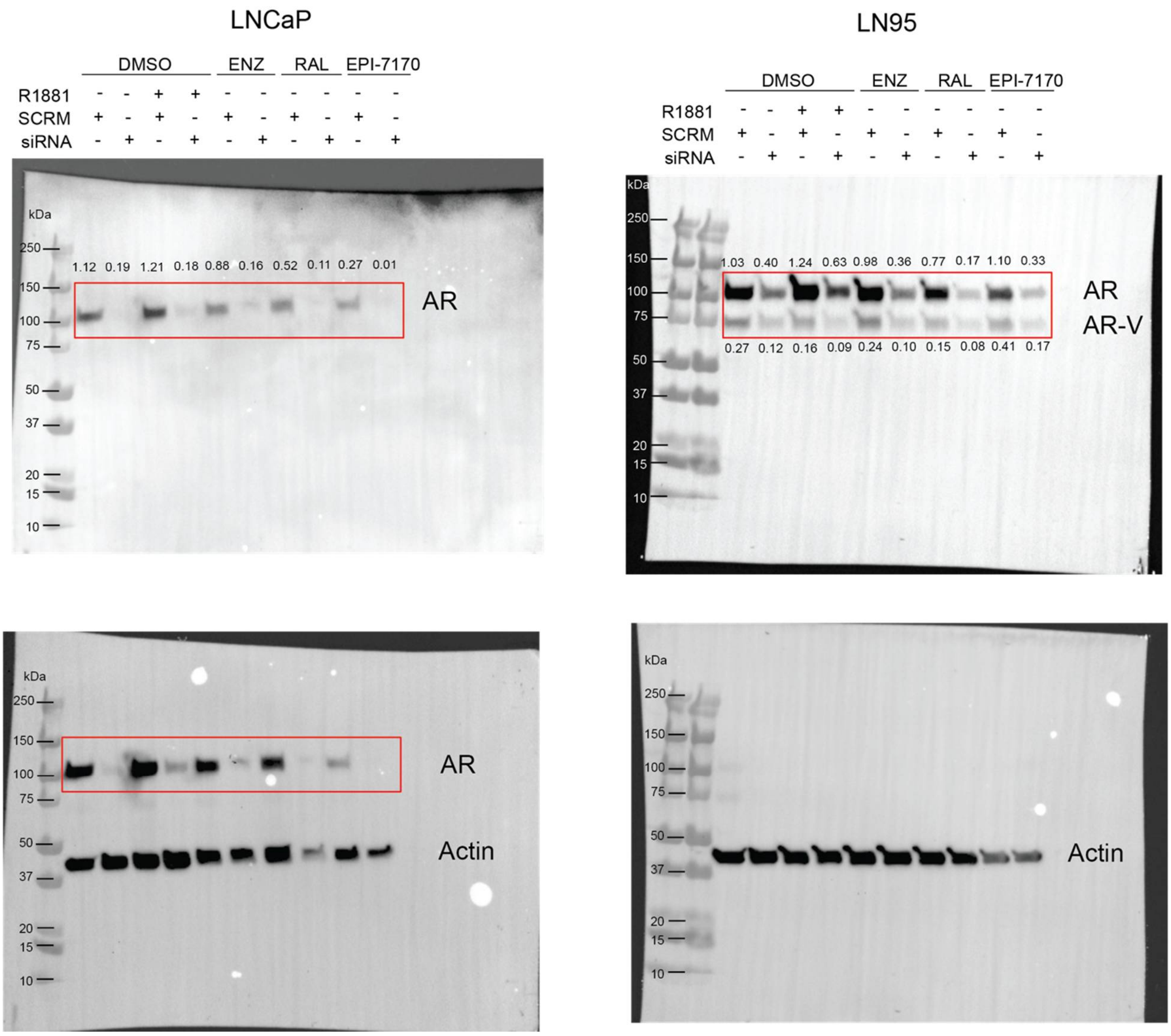

Figure S2. : (related to Figure 4) Whole uncut Western blots are shown. The intensity of each band is normalized to that of actin and the ratios are indicated on the figure. Red rectangles indicate the proteins of interest.

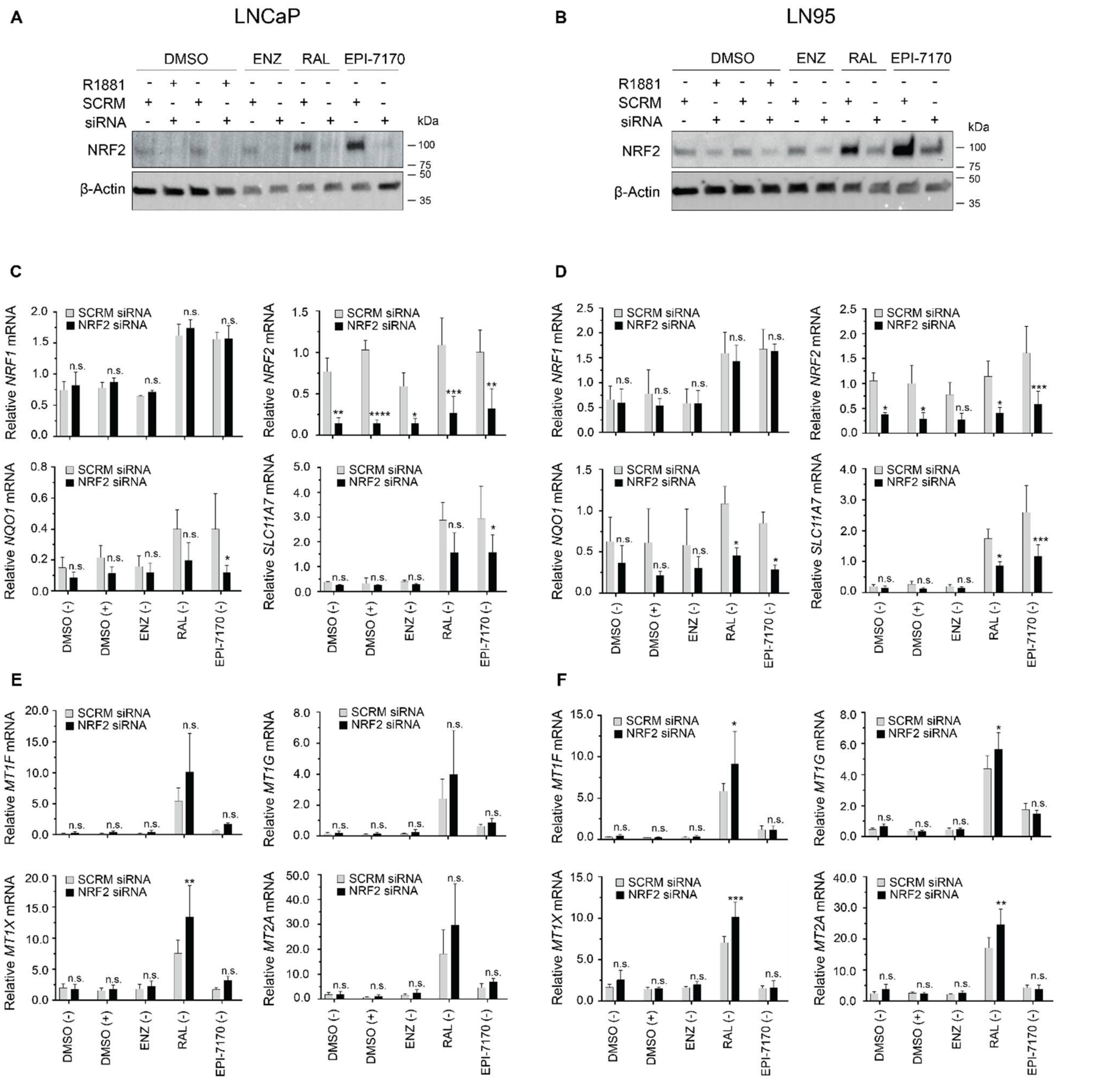

Figure S3. Ralaniten induced expression of metallothionein is independent of levels of NRF2. (**A**) Western blot analysis of levels of NRF2 proteins in LNCaP and (**B**) LN95 cells transfected with scramble (SCRM, control) or NRF2 siRNA (10 nM) for 24 hours and treated with DMSO, enzalutamide (5 μM), ralaniten (35 μM), or EPI-7170 (5 μM) in the presence or absence of 1 nM R1881. (**C**) Transcript levels of *NRF1*, *NRF2*, *NQO1*, and *SLC11A7* were normalized to transcript levels of *SDHA* in LNCaP and (**D**) LN95 cells treated with DMSO, enzalutamide (5 μM), ralaniten (35 μM), or EPI-7170 (5 μM) in the presence (+) or absence (-) of 1 nM R1881. (**E**) Transcript levels of *MT1F*, *MT1G*, *MT1X*, and *MT2A* were normalized to transcript levels of *SDHA* from LNCaP and (**F**) LN95 cells treated with DMSO, enzalutamide (5 μM), ralaniten (35 μM), or EPI-7170 (5 μM) in the presence (+) or absence (−) of 1 nM R1881. Data presented as mean ± SD and analyzed by two-way ANOVA with Sidak's test applied post hoc, $n = 3$ independent experiments. $^{*}p < 0.05$; $^{**}p < 0.01$; $^{***}p < 0.001$; $^{\#}p < 0.0001$; n.s., not significant. **ENZ**, enzalutamide; **RAL**, ralaniten; **+**, R1881, **-**, EtOH.

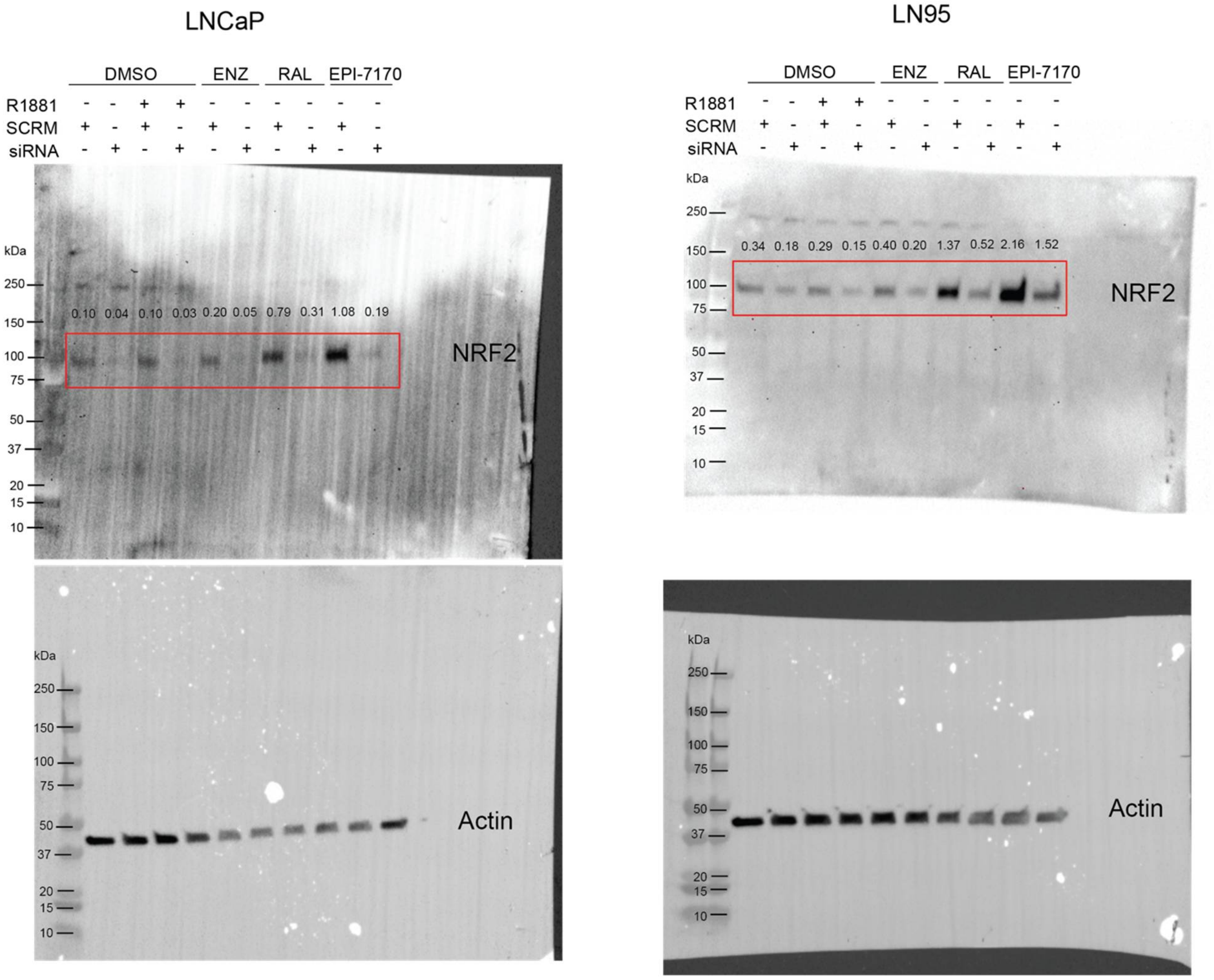

Figure S4. (related to Supplementary Figure S3). Whole uncut Western blots are shown. The intensity of each band is normalized to that of actin and the ratios are indicated on the figure. Red rectangles indicate the proteins of interest.

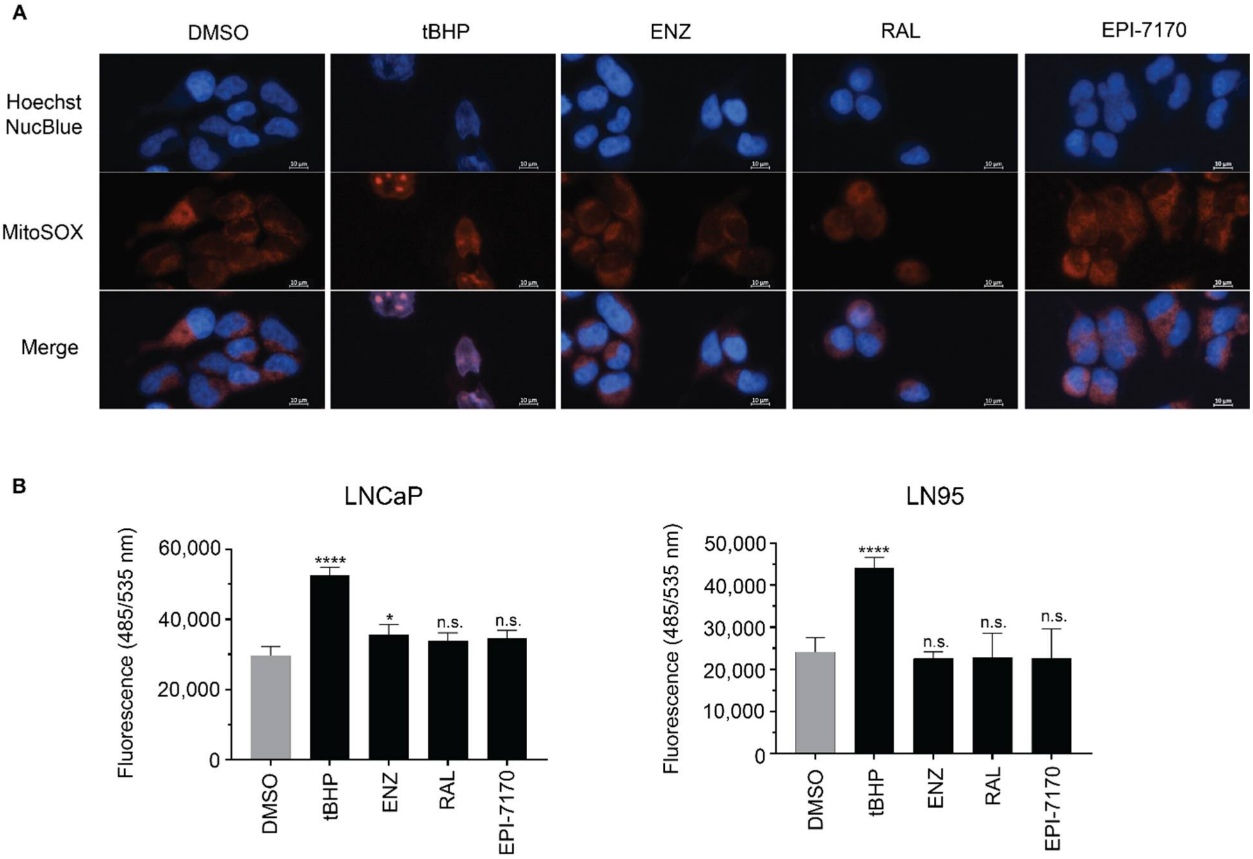

Figure S5. Neither ralaniten nor EPI-7170 induce ROS generation. (**A**) LN95 cells were treated tBHP (75 μM), enzalutamide (5 μM) ralaniten (35 μM) EPI-7170 (5 μM) or DMSO vehicle for 4 hrs prior to staining with 5 μM MitoSOX Red. Representative image from $n = 3$ experiments. Bars = 10 μm. (**B**) Detection of total cellular ROS in LNCaP and LN95 cells was detected using DCFDA staining. Cells were stained with with DCFDA/H2DCFDA (20 μM) for 30 minutes followed by treatment with tBHP (75 μM), enzalutamide (5 μM), ralaniten (35 μM) or EPI-7170 (5 μM) or DMSO vehicle for 4 hours. Fluorescence was measured with excitation at 485 nm and emission at 535 nm. Data presented as mean ± SD and analyzed by two-way ANOVA with Sidak's test applied *post hoc*, $n=3$ independent experiments. *$p < 0.05$; **$p < 0.01$; ***$p < 0.001$; $^{\#}p < 0.0001$; n.s., not significant. ENZ, enzalutamide; RAL, ralaniten; tBHP, tert-butyl hydroperoxide.

Table S1. Gene-specific primer sequences for qRT-PCR experiments.

Target	Direction	Sequence
	FWD	5'-AGGAACTCGATCCTATCATTGC-3'
AR	REV	5'-CTGCCATCATTTCCGGAA-3'
	FWD	5'-CCATCTTGTCGTCTTCGGAAATGTTAT-3'
AR-V7	REV	5'-TTTGAATGAGGCAAGTCAGCCTTTCT-3'
	FWD	5'-CGCAGGATATACGCCAACAT-3'
FKBP5	REV	5'-GAAGTCTTCTTGCCCATTGC-3'
	FWD	5'-TCATCCTGTCTCGGATTGTG-3'
PSA	REV	5'-ATATCGTAGAGGGGGTGTGG-3'
	FWD	5'-ACAGAGAGACATGTACAAACCTGG-3'
MT1F	REV	5'-GAATGTAGCAAATGGGTCAAGGTG-3'
	FWD	5'-ATAGAGTGACCCGTAAAATCCAGG-3'
MT1G	REV	5'-TAGCAAAGGGGTCAAGATTGTAGC-3'
	FWD	5'-GTGTTTTCCTCTTGATCGGGAACTC-3'
MT1X	REV	5'-TCCATTTCGAGGCAAGGAGAAG-3'
	FWD	5'-AGATGTAAAGAACGCGACTTCCAC-3'
MT2A	REV	5'-AATATAGCAAACGGTCACGGTCAG-3'
	FWD	5'-CACCCTGTACGTTATCTTCTAGCTC-3'

MTF1	REV	5'-CAGTTTCCTTACCACCTCCTAAGTC-3'
	FWD	5'-AGAAGCAGAGAAGTGCAG-3'
MT3	REV	5'-GGGATTTATTGTCATTCCTCC-3'
	FWD	5'-CAGCATGTGTTACCAAGCTGT-3'
SDHA	REV	5'-CGTGTCGTAGAAATGCCACCT-3'
	FWD	5'-GGTGAAGATTTGGAGGATTTG-3'
NRF1	REV	5'-TCAGATCTATGTCCTCTTTGG-3'
	FWD	5'-CGTTTGTAGATGACAATGAGG-3'
NRF2	REV	5'-AGAAGTTTCAGGTGACTGAG-3'
	FWD	5'-TTTGTGGGTCTGTAGAAATG-3'
NQO1	REV	5'-AGTATCCACAATAGCTGACG-3'
	FWD	5'-GGTTATTCTATGTTGCGTCTC-3'
SLC11A7	REV	5'-AATAACAGCTGGTAGAGGAG-3'
